# Christian Science and the Law

**Published:** 1899-01

**Authors:** 


### "Christian Science and the Law."

It is so rare a thing for the daily press to discuss a medical topic in anything like a professional and unbiased manner, that for this very reason the leading editorial of one of our morning dailies (the *Commercial-Tribune,* Monday, December 12, 1898), bearing the above title is entitled to more than cursory reading. Those who read it will remember that it was *apropos* of the McDowell case, in which the prosecutor insisted that the proper charge against Miss Evans was manslaughter.

At this time, when the newspapers are so often compelling their long-suffering constituents to peruse with bated breath the miraculous cures of adherents to this or that "ology," "pathy" or "ism," of cases heralded in large type, "Medical Men Puzzled," followed by the cry in softer ink, "Quackopathy to the Rescue," it is rather refreshing to read an article in one of them which, while not eulogizing the medical profession, is certainly dealing with one of the enemies of scientific advance, if one might be allowed the expression, without gloves. Space does not permit a complete reproduction of the rather lengthy editorial, but a few extracts may prove of interest.

In speaking of political liberty, the writer divided it into two parts: "First, matters of thought and action shall be absolutely divorced, and law shall take cognizance of the latter only; second, the source of authoritv (corrected, of course, by the pardoning power of the Executive), is simply and solely the will of the majority." "The will of the majority" in this day of ward bosses, city bosses, State bosses, may seem at first sight to be a very crude and uncertain kind of liberty, but the great upheavals in political circles from time to time, often an apparent complete change in sectional politics, shows that the President who made the remark about "fooling the people" was not far wrong. The first division, the separation of thought, or better, of speech, from action, even more forcibly strikes the keynote of political liberty.

It is the old story of freedom of speech, freedom of worship, upon which this country was first founded, and it is this, combined with observance and enforcement of law, that has made government "by the people" possible. In the words of the editorial, "An Anglo-Saxon * * * may think what he pleases, he may plead and preach what he pleases; so long as he does not take that last step which crosses the middle line between thought and action, the law must let him alone. He may go alone to convert the whole nation to Mohammedanism, and the law stands idly by. Let him wear a red necktie, when the law has forbidden it, and if we have robust Governors he will be in jail before night."

And the whole of this lengthy preamble is but to bring out the pith of the article more forcibly, "Christian Science and the Law;" and again the words of the editorial impress most strongly: "If Christian Science wants to bring the nation to its way of thinking, why, let it plead, preach, harangue, write, pamphletize, to its heart's content. When, at last, it shall win over the majority and get itself recognized on the statute book, then let it go in peace and practice what it pleases. But until then let the courts do their full duty. When a Christian scientist has contributed to the death of a patient let them consider his motives, no more, no less, than they would consider those of a physician who willfully kept from his patient an antidote to poison. *Prosecutor Lueders was quite right when he said, in the McDowell case, that the proper charge against Miss Evans was manslaughter.*"

This is indeed a most fair and practical way of looking at the question. If only the juries convened to try these self-styled physicians and scientists can be made to see the matter in the same light, these various colleges of "ologies" and "pathies" will die a natural death. But a few days ago, a man, a chronic alcoholic, afflicted with delirium tremens, died some hours after the injection of a half-grain of morphia in divided doses. An inquest had to be held, on the outcry of the afflicted wife, to acquit the physician who had given the injections of the charge of killing his patient, when in reality the physician was doing probably the only thing in his power that would save the man's life. It would be well for the community if a few inquests were held where the "scientists," etc., sat idly by and allowed their victims—it would be folly to call them patients—die without effort being made to restore them. We would respectfully ask, how is a patient in coma or delirium going to exercise his will power to persuade himself that there is nothing the matter with him?

We earnestly hope the disease of the *Commercial* is an acute infectious one, with several times the virulence of the present epidemic, and that no efforts will be made to prevent the contagium spreading among its contemporaries.—Editorial in *Lancet-Clinic.*

